# Central Treatment of Ketone Body in Rainbow Trout Alters Liver Metabolism Without Apparently Altering the Regulation of Food Intake

**DOI:** 10.3389/fphys.2019.01206

**Published:** 2019-09-18

**Authors:** Sara Comesaña, Cristina Velasco, Marta Conde-Sieira, Cristina Otero-Rodiño, Jesús M. Míguez, José L. Soengas

**Affiliations:** Laboratorio de Fisioloxía Animal, Departamento de Bioloxía Funcional e Ciencias da Saúde, Facultade de Bioloxía and Centro de Investigación Mariña-CIM, Universidade de Vigo, Vigo, Spain

**Keywords:** β-hydroxybutyrate, ketone body, food intake, hypothalamus, hindbrain, liver

## Abstract

We hypothesize that the presence in fish brain of a ketone body (KB) like β-hydroxybutyrate (BHB) alters energy homeostasis through effects on food intake and peripheral energy metabolism. Using rainbow trout (*Oncorhynchus mykiss*) as a model, we intracerebroventricularly (ICV) administered 1 μl 100 g^–1^ body mass of saline solution alone (control) or containing 0.5 μmol of BHB. In a fist set of experiments, BHB did not affect food intake 6 and 24 h after treatment. In a second set of experiments, we evaluated 6 h after ICV BHB treatment changes in parameters putatively related to food intake control in brain areas (hypothalamus and hindbrain) involved in nutrient sensing and changes in energy metabolism in liver. The absence of changes in food intake might relate to the absence of major changes in the cascade of events from the detection of KB through ketone-sensing mechanisms, changes in transcription factors, and changes in the mRNA abundance of neuropeptides regulating food intake. This response is different than that of mammals. In contrast, central administration of BHB induced changes in liver energy metabolism suggesting a decreased use of glucose and probably an enhanced use of amino acid and lipid. These responses in liver are different to those of mammals under similar treatments but comparable to those occurring in fish under food deprivation conditions.

## Introduction

During food deprivation ketone bodies (KB), β-hydroxybutyrate (BHB) and acetoacetate synthetized, mainly in liver, from fatty acids are used as a metabolic fuel in the brain as demonstrated in mice and rat (Paoli et al., 2015; Carneiro et al., 2016a; Le Foll and Levin, 2016; Rojas-Morales et al., 2016). KB are also synthesized in brain astrocytes (Le Foll and Levin, 2016). Since KB are produced as an effect of food deprivation, they might signal a condition of energy shortage and subsequently increase food intake to maintain energy homeostasis (Carneiro et al., 2016a; Rojas-Morales et al., 2016). This hypothesis is supported by studies in which central infusion of BHB increased food intake in rat (Iwata et al., 2011) and mice (Carneiro et al., 2016a, b). However, subcutaneous injection of BHB or feeding a ketogenic diet reduces food intake in rat (Langhans et al., 1983) and mice (Paoli et al., 2015; Le Foll and Levin, 2016). A regulatory response to the central presence of KB can be also elicited in peripheral tissues via the autonomic nervous system in rat (Park et al., 2011) and mice (Carneiro et al., 2016a, b).

The detection of changes in KB levels in brain is therefore important for the regulation of food intake and peripheral metabolism. Not surprisingly, brain areas involved in nutrient sensing such as hypothalamus show the highest utilization rates of BHB (Laeger et al., 2010). Several mechanisms could be involved in this ketone-sensing capacity. The metabolism of KB might be involved since inhibition of ketogenesis in astrocytes with hymeglusin reversed the decrease in food intake observed in rats fed a ketogenic diet (Le Foll et al., 2015). In this mechanism, a rise in BHB levels would result in increased catabolism and decreased synthesis through changes in the activity of key enzymes involved in rate-limiting steps of ketogenesis and ketolysis such as β-hydroxybutyrate dehydrogenase (β-HBDH), acetyl-CoA C-acetyltransferase (ACAT), succinyl-CoA:3-ketoacid CoA transferase (SCOT), and hydroxymethylglutaryl-CoA synthase (HMGCS). The transport of KB can be also a putative mechanism of ketone-sensing. Several monocarboxylate transporters (MCTs) carry BHB across the blood–brain barrier, and their expression can be regulated in mice by brain BHB uptake (Laeger et al., 2010; Newman and Verdin, 2017), and also by peroxisome proliferator-activated receptor α (PPARα) (Schutkowski et al., 2014). Moreover, inhibition of MCT1 suppressed BHB-stimulated AMP-activated protein kinase (AMPK) phosphorylation in mice (Laeger et al., 2012). Thus, AMPK might also mediate the response to BHB. Accordingly, KB modulate the phosphorylation status of AMPK in a time-dependent way in mice (Laeger et al., 2012).

In teleost fish, several studies carried out in recent years in rainbow trout (*Oncorhynchus mykiss*) demonstrated in brain areas (especially hypothalamus and hindbrain) the presence and functioning of nutrient sensors (for glucose, fatty acid, and amino acid) involved in the regulation of food intake (Conde-Sieira and Soengas, 2017; Delgado et al., 2017; Soengas et al., 2018). There are no studies in fish relating the presence of KB in the brain to food intake control and peripheral energy metabolism. Available studies demonstrated that food deprivation enhanced ketogenesis in liver of several fish species including rainbow trout (Furné et al., 2012) with a rise in KB content in brain, as demonstrated in rainbow trout (Soengas et al., 1998) and Atlantic salmon (Soengas et al., 1996). The time period in which these increases occur is considerably longer than in mammals whereas the magnitude of the change is lower. Furthermore, the oxidative use of BHB in rainbow trout brain is considerably lower than that of other fuels like glucose or lactate (Soengas et al., 1998). This is remarkably different than in mammals in which oxidative rates of BHB are similar to those of glucose (Laeger et al., 2010; Paoli et al., 2015; Rojas-Morales et al., 2016). During food deprivation in rainbow trout brain (Soengas et al., 1998) despite a rise in BHB oxidative capacity, rates were still lower than those of glucose. It seems therefore that KB metabolism is less important in the brain of teleost fish than in mammals.

We therefore hypothesize that the impact of a central treatment with KB in teleost fish might have a reduced effect compared with mammals regarding the necessity of activating food intake. Accordingly, in a first set of experiments, we observed that intracerebroventricular (ICV) treatment with BHB did not affect food intake of rainbow trout. In a second set of experiments, we aimed to assess the reasons behind this lack of response. Thus, we carried out a similar ICV treatment and evaluated in brain areas involved in food intake control in fish, such as hypothalamus and hindbrain (Soengas et al., 2018), their metabolic status, the response of putative ketone-sensing mechanisms (not characterized before in fish), and the response of parameters related to food intake control such as integrative sensors, transcription factors, and mRNA abundance of neuropeptides. The presence of KB in the brain may also impact peripheral energy metabolism through sympathetic and parasympathetic systems that innervate gastrointestinal tract and liver in the same species (Seth and Axelsson, 2010). We hypothesize that changes occurring in liver of rainbow trout after central treatment with BHB should be of a lower magnitude than those of mammals. However, considering the relatively lower importance of glucose metabolism in fish compared with that of amino acid and lipid (Polakof et al., 2011, 2012), these changes may affect pathways different than those altered in mammals. Therefore, we assessed in liver changes in signaling pathways as well as in parameters related to main pathways of glucose, fatty acid, and amino acid metabolism.

## Materials and Methods

### Fish

Rainbow trout were obtained from a local fish farm (A Estrada, Spain). Fish were maintained for 1 month in 100 l tanks under laboratory conditions and 12L:12D photoperiod (lights on at 08:00 h, Lights off at 20:00 h) in dechlorinated tap water at 15°C. Fish were fed once daily (09.00 h) to satiety with commercial dry fish pellets (Dibaq-Diproteg SA, Spain; proximate food analysis was 48% crude protein, 14% carbohydrates, 25% crude fat, and 11.5% ash; 20.2 MJ/kg of feed). The experiments described comply with the ARRIVE Guidelines, and were carried out in accordance with the guidelines of the European Union Council (2010/63/UE), and of the Spanish Government (RD 53/2013) for the use of animals in research, and were approved by the Ethics Committee of the Universidade de Vigo.

### Experimental Design

Following 1-month acclimation period, fish were randomly assigned to 100 l experimental tanks. Fish were fasted for 24 h before treatment to ensure basal levels of hormones involved in metabolic control were achieved. On the day of experiment, fish were lightly anaesthetized with 2-phenoxyethanol (Sigma, 0.02% v/v), and weighed. ICV administration was performed as previously described (Polakof and Soengas, 2008). Briefly, fish were placed on a plexiglass board with Velcro straps adjusted to hold them in place. A 291/2 gauge needle attached through a polyethylene cannula to a 10 μl Hamilton syringe was aligned with the 6th preorbital bone at the rear of the eye socket, and from this point the syringe was moved through the space in the frontal bone into the third ventricle. The plunger of the syringe was slowly depressed to dispense 1 μl 100 g^–1^ body mass of saline solution alone (control) or containing 0.5 μmol of BHB. The dose was calculated with the concentration of approx. 2.5 μmol of acetoacetate g^–1^ wet weight measured in brain of the same species after 5 days of food deprivation (Figueroa et al., 2000), a brain size of approx. 0.2 g, and assuming an easy conversion of acetoacetate into BHB in brain.

In a first set of experiments, fish of 75.8 ± 2.7 g size were randomly distributed in tanks to be used for the assessment of food intake. This was registered for 3 days before treatment (to evaluate basal level of food intake) and then 6 and 24 h after ICV treatment with saline solution alone (control, *n* = 10 fish) or containing BHB (*n* = 10 fish). These time periods were selected based on prior studies in the same species describing changes in food intake after ICV treatment with different nutrients (Conde-Sieira and Soengas, 2017; Delgado et al., 2017). After feeding, uneaten food remaining at the bottom of the conical tanks was withdrawn, dried and weighed, and this value was used to calculate the amount of food consumed by all fish in each tank, as the difference from the feed offered (Polakof et al., 2008a, b). The experiment was repeated three times, and results are shown as the mean ± SEM of 3 experiments (*N* = 3) with *n* = 10 fish per treatment in each tank per experiment.

In a second set of experiments, fish of 88.8 ± 1.9 g size were randomly distributed to be ICV injected with saline solution alone (control, *n* = 22) or containing BHB (*n* = 22) with the same concentration described above. After 6 h, again a time period known to induce in the same species changes in central and peripheral metabolism and parameters related to food intake control (Conde-Sieira and Soengas, 2017; Delgado et al., 2017), fish were anaesthetized with 2-phenoxyethanol (Sigma, 0.02% v/v) and sampled sequentially following the same order of injection. Blood was collected by caudal puncture with ammonium-heparinized syringes, and plasma samples were obtained after blood centrifugation, deproteinized immediately (using 0.6 M perchloric acid) and neutralized (using 1 M potassium bicarbonate) before freezing on dry ice and storage at –80°C until further assay. Fish were sacrificed by decapitation, and hypothalamus, hindbrain and liver were dissected, snap-frozen, and stored at –80°C. Ten fish per group were used to assess enzyme activities and metabolite levels, six fish per group were used for the assessment of mRNA levels by qRT-PCR, whereas the remaining six fish per group were used to assess changes in the levels of proteins by Western blot.

### Assessment of Metabolite Levels and Enzyme Activities

Levels of glucose, lactate and fatty acid in plasma were determined enzymatically using commercial kits (Spinreact, Barcelona, Spain, for glucose and lactate and Wako Chemicals, Neuss, Germany, for fatty acid). Acetoacetate and BHB levels were assessed following enzymatic methods described by Mellanby and Williamson (1974) and Williamson and Mellanby (1974), respectively.

Samples used to assess tissue metabolite levels were homogenized immediately by ultrasonic disruption in 7.5 vols of ice-cooled 0.6 M perchloric acid, and neutralized with 1 M potassium bicarbonate. The homogenate was centrifuged (10,000 × *g*), and the supernatant used to assay tissue metabolites. Levels of glucose, lactate, fatty acid, acetoacetate, and BHB were determined in brain areas as described above for plasma samples. In liver, the same metabolites were assessed as well as levels of glycogen, triglyceride, and α-amino acid. Tissue glycogen levels were assessed as described by Keppler and Decker (1974). Tissue total α-amino acid levels were determined colorimetrically using the nynhydrin method (Moore, 1968) with alanine as standard.

Samples for enzyme activities were homogenized by ultrasonic disruption with 9 vols of ice-cold-buffer consisting of 50 mM Tris (pH 7.6), 5 mM EDTA, 2 mM 1,4-dithiothreitol, and a protease inhibitor cocktail (Sigma, St. Louis, MO, United States). The homogenate was centrifuged (1,000 × *g*) and the supernatant used immediately for enzyme assays. Enzyme activities were determined using a microplate reader INFINITE 200 Pro (Tecan, Männedorf, Switzerland). Reaction rates of enzymes were determined by the decrease or increase in absorbance of NADH at 340 nm, of acetoacetyl-CoA at 300 nm in the case of ACAT and SCOT, and in the case of CPT-1 activity, of 5,5′-dithiobis (2-nitrobenzoic acid)-CoA complex at 412 nm. The reactions were started by the addition of supernatant (10–20 μl) at a pre-established protein concentration, omitting the substrate in control wells (final volume 180–285 μl), and allowing the reactions to proceed at 20°C for pre-established times (3–30 min). Enzyme activities were normalized to protein levels (mg). Protein was assayed in triplicate in homogenates using microplates according to the bicinchoninic acid method with bovine serum albumin (Sigma) as standard. Enzyme activities were assessed at maximum rates determined by preliminary tests.

Acetyl-CoA C-acetyltransferase (*EC* 2.3.1.9) and SCOT (*EC* 2.8.3.5) activities were assessed by adaptation of available methods (Williamson et al., 1971). ACAT activity was assessed in a Tris buffer (50 mM, pH 8.5) containing 5 mM MgCl_2_, 0.1 mM coenzyme A, and 90 μM acetoacetyl-CoA (omitted for controls). SCOT activity was assessed in a Tris buffer (50 mM, pH 8) containing 5 mM MgCl_2_, 4 mM iodoacetamide, 0.2 mM acetoacetate, and 2 μM succinyl-CoA (omitted for controls). β-HBDH (*EC* 1.1.1.30), glucokinase (GCK, *EC* 2.7.1.2), pyruvate kinase (PK, *EC* 2.7.1.40), phosphoenolpyruvate carboxykinase (PEPCK, *EC* 4.1.1.32), glycogen synthase (GSase, *EC* 1.1.1.35), glucose 6-phosphatase (G6Pase, *EC* 3.1.3.9), carnitine palmitoyl transferase type 1 (CPT-1, *EC* 2.3.1.21), fatty acid synthase (FAS, *EC* 2.3.1.85), glutamate dehydrogenase (GDH, *EC* 1.4.1.2), and alanine transaminase (ALT, *EC* 2.6.1.2) activities were determined using previously described methods (Soengas et al., 1998; Conde-Sieira et al., 2019). Total GSase activities were measured with 5 mM glucose 6-phosphate (G6P) present, and GSase a activities were estimated lacking G6P; the ratio of GSase activities without and with G6P multiplied by 100 represents the percentage of total GSase (a + b) in the active form (% GSase a).

### mRNA Abundance Analysis by Real-Time Quantitative PCR

Total RNA was extracted using Trizol reagent (Life Technologies, Grand Island, NY, United States) and subsequently treated with RQ1-DNAse (Promega, Madison, WI, United States). Two microgram total RNA were reverse transcribed using Superscript II reverse transcriptase (Promega) and random hexamers (Promega) to obtain approx. 20 μl. Gene expression levels were determined by real-time quantitative (RT qPCR) using the iCycler iQ (BIO-RAD, Hercules, CA, United States). Analyses were performed on 1 μl cDNA using MAXIMA SYBR Green qPCR Mastermix (Life Technologies), in a total PCR reaction volume of 15 μl, containing 50–500 nM of each primer. Therefore, we assessed mRNA abundance of transcripts related to (1) ketone-sensing such as β-hydroxybutyrate dehydrogenase (*bdh1*), hydroxymethylglutaryl-CoA synthase (*hmcgs2*), monocarboxylate transporter 1 (*slc16a1*), and monocarboxylate transporter 2 (*slc16a7*); (2) food intake control such as the transcription factors brain homeobox transcription factor (*bsx*), cAMP response-element-binding protein (*creb1*), forkhead boxO1 (*foxo1*), and peroxisome proliferator-activated receptor type α (*ppara*), and the neuropeptides agouti-related protein 1 (*agrp1*), neuropeptide Y (*npy*), pro-opio melanocortin a1 (*pomca1*), and cocaine- and amphetamine-related transcript (*cartpt*); (3) integrative sensors such as mechanistic target of rapamycin (*mtor*), and sirtuin 1 (*sirt1*); (4) glucose metabolism such as fructose 1,6-bisphosphatase (*fbp1*), glucose 6-phosphatase (*g6pc*), glucokinase (*gck*), glucose facilitated transporter type 2 (*slc2a2*), glycogen synthase (*gys1*), phosphoenolpyruvate carboxykinase (*pck1*); (5) K^+^_ATP_ channel such as inward rectifier K^+^ channel pore type 6 (*kcnj11*) and sulfonylurea receptor 1 (*abcc8*); (6) fatty acid metabolism such as carnitine palmitoyl transferase 1 types a and c (*cpt1a* and *cpt1c*), fatty acid synthetase (*fasn*), fatty acid translocase (*cd36*), fatty acid transport protein 1 (*slc27a1*), and 3-hydroxyacyl-CoA dehydrogenase (*hadh*); (7) lactate metabolism such as lactate dehydrogenase chain a (*ldha*) and lactate dehydrogenase chain b (*ldhb*); and, (8) amino acid metabolism such as alanine transaminase (*gpt*) and glutamate dehydrogenase (*glud1*). Sequences of the forward and reverse primers used for each transcript expression are shown in Table 1. Most transcripts were measured using previously described primers in the same species (Table 1) with the exception of *bdh1* and *hmgcs2*. For these transcripts, new primers were designed using Primer3 software^1^ from sequences available in GenBank (*bdh1*, XM_021574638.1; *hmgcs2*, XM_021601620.1). The DNA products obtained by RT-PCR using total RNA isolated were purified and ligated into the pGEM T-easy vector (Promega, Madison, WI, United States) and transformation was used to clone the resulting plasmid into *Escherichia coli* JM109 cells. Single white colonies were picked up and cultured in LB medium with 100 μg ml^–1^ ampicillin. The recombinant plasmid was isolated from the recombinant clones and subjected to DNA sequencing by the Sanger method in Centro de Apoio Científico-Tecnolóxico (CACTI) of Universidade de Vigo. DNA sequences were used for homology search using blast. Relative quantification of the target gene transcript was done using *actb* (β-actin) and *eef1a1* (elongation factor 1α) gene expression as reference, which were stably expressed in this experiment. Thermal cycling was initiated with incubation at 95°C for 90 s using hot-start iTaq DNA polymerase activation followed by 35 cycles, each one consisting of heating at 95°C for 20 s, and specific annealing and extension temperatures for 20 s. Following the final PCR cycle, melting curves were systematically monitored (55°C temperature gradient at 0.5°C/s from 55 to 94°C) to ensure that only one fragment was amplified. Samples without reverse transcriptase and samples without RNA were run for each reaction as negative controls. Only efficiency values between 85 and 100% were accepted (The *R*^2^ for all genes assessed was higher than 0.985). Relative quantification of the target gene transcript with the *actb* and *eef1a1* reference gene transcripts was made following the method Pfaffl (2001).

**TABLE 1:** Nucleotide sequences of the PCR primers used to evaluate mRNA abundance by RT-PCR (qPCR).

**Gene**	**Forward primer**	**Reverse primer**	**Annealing**	**Data base**	**Accession number**
			**temperature (°C)**		
*abcc8*	CGAGGACTGGCCCCAGCA	GACTTTCCACTTCCTGTGCGTCC	62	Sigenae	tcce0019d.e.20_3.1.s.om.8
*actb*	GATGGGCCAGAAAGACAGCTA	TCGTCCCAGTTGGTGACGAT	59	GenBank	NM_ 001124235.1
*agrp1*	ACCAGCAGTCCTGTCTGGGTAA	AGTAGCAGATGGAGCCGAACA	60	GenBank	CR376289
*bdh1*	TCGCAATATGGCTGAGGTCAA	ACACCATCCGTCCTTTTGAGG	63	GenBank	XM_021574636.1
*bsx*	CATCCAGAGTTACCCGGCAAG	TTTTCACCTGGGTTTCCGAGA	60	GenBank	MG310161
*cartpt*	ACCATGGAGAGCTCCAG	GCGCACTGCTCTCCAA	60	GenBank	NM_001124627
*cd36*	CAAGTCAGCGACAAACCAGA	ACTTCTGAGCCTCCACAGGA	62	DFCI	AY606034.1
*cpt1a*	TCGATTTTCAAGGGTCTTCG	CACAACGATCAGCAAACTGG	55	GenBank	AF327058
*cpt1c*	CGCTTCAAGAATGGGGTGAT	CAACCACCTGCTGTTTCTCA	59	GenBank	AJ619768
*creb1*	CGGATACCAGTTGGAGGAGGA	AGCAGCAGCACTCGTTTAGGC	60	GenBank	MG310160
*eef1a1*	TCCTCTTGGTCGTTTCGCTG	ACCCGAGGGACATCCTGTG	59	GenBank	AF498320
*fasn*	GAGACCTAGTGGAGGCTGTC	TCTTGTTGATGGTGAGCTGT	59	Sigenae	tcab0001c.e.06 5.1.s.om.8
*fbp1*	GCTGGACCCTTCCATCGG	CGACATAACGCCCACCATAGG	59	GenBank	AF333188
*foxo1*	AACTCCCACAGCCACAGCAAT	CGATGTCCTGTTCCAGGAAGG	60	GenBank	MG310159
*g6pc*	CTCAGTGGCGACAGAAAGG	TACACAGCAGCATCCAGAGC	55	Sigenae	cay0019b.d.18_3.1.s.om.8.1-1693
*gck*	GCACGGCTGAGATGCTCTTTG	GCCTTGAACCCTTTGGTCCAG	60	GenBank	AF053331
*glud1*	TGCTGACACCTATGCCAACAC	CCTGGCTGATGGGCTTACC	58	GenBank	AJ556997
*gpt*	GGGAGTAGTTTGGGTGCGTACA	CGTCCTGCCGCACACA	60	GenBank	CA373015
*gys1*	CGTGGTGAGAGGAAGGAACTGAGC	CCGTTGAGACCGTGGAGACA	59	GenBank	BT073381.1
*hadh*	GGACAAAGTGGCACCAGCAC	GGGACGGGGTTGAAGAAGTG	59	Sigenae	tcad0001a.i.15 3.1.om
*hmgcs2*	TAGCTCCTGGGACGGTCGTTA	GGCACTTCCTGTGGCGTAAAC	60	GenBank	XM_021601620.1
*kcnj11*	TTGGCTCCTCTTCGCCATGT	AAAGCCGATGGTCACCTGGA	60	Sigenae	CA346261.1.s.om.8:1:773:1
*ldha*	TCCGTGCTAGGTGCTGTGTGA	ACACCACTGCTCCCCGACAG	60	Sigenae	BE859108.p.om.7
*ldhb*	TTCCACGTGAGGCTATAATGGACA	TGGCACAGGGGGCTCTTTAC	60	Sigenae	CA357739.p.om.7
*mtor*	ATGGTTCGATCACTGGTCATCA	TCCACTCTTGCCACAGAGAC	60	GenBank	EU179853
*npy*	CTCGTCTGGACCTTTATATGC	GTTCATCATATCTGGACTGTG	58	GenBank	NM_001124266
*pck1*	GTTGGTGCTAAAGGGCACAC	CCCGTCTTCTGATAAGTCCAA	59	GenBank	AF246149
*pomca1*	CTCGCTGTCAAGACCTCAACTCT	GAGTTGGGTTGGAGATGGACCTC	60	Tigr	TC86162
*ppara*	CTGGAGCTGGATGACAGTGA	GGCAAGTTTTTGCAGCAGAT	55	GenBank	AY494835
*sirt1*	GCTACTTGGGGACTGTGACG	CTCAAAGTCTCCGCCCAAC	57	GenBank	EZ774344.1
*slc16a1*	AGGCTTGGGACTGGCATTCA	AGCCACGCAGCAGTTGAACA	60	Sigenae	CX251492.p.om.7
*slc16a7*	CGACCACTTGTTTGGGGACTGTC	TATGTCCTTGGCATAGGCCGTC	60	GenBank	KF032406.1
*slc27a1*	AGGAGAGAACGTCTCCACCA	CGCATCACAGTCAAATGTCC	60	DFCI	CA373015
*slc2a2*	GTGGAGAAGGAGGCGCAAGT	GCCACCGACACCATGGTAAA	59	GenBank	AF321816

**abcc8*, sulfonylurea receptor 1; *actb*, β-actin; *agrp1*, agouti-related protein 1; *bdh1*, β-hydroxybutyrate dehydrogenase; *bsx*, brain homeobox transcription factor; *cartpt*, cocaine- and amphetamine-related transcript; *cd36*, fatty acid translocase; *cpt1a*, carnitine palmitoyl transferase type 1a; *cpt1c*, carnitine palmitoyl transferase type 1c; *creb1*, cAMP response-element-binding protein; *eef1a1*, elongation factor 1α; *fasn*, fatty acid synthetase; *fbp1*, fructose 1,6-bisphosphatase; *foxo1*, forkhead boxO1; *g6pc*, glucose 6-phosphatase; *gck*, glucokinase; *glud1*, glutamate dehydrogenase; *gpt*, alanine transaminase; *gys1*, glycogen synthase; *hadh*, 3-hydroxyacyl-CoA dehydrogenase; *hmgcs2*, hydroxymethylglutaryl-CoA synthase; *kcnj11*, inward rectifier K^+^ channel pore type 6; *ldha*, lactate dehydrogenase chain a; *ldhb*, lactate dehydrogenase chain b; *mtor*, mechanistic target of rapamycin; *npy*, neuropeptide Y; *pck1*, phosphoenolpyruvate carboxykinase; *pomca1*, pro-opio melanocortin a1; *ppara*, peroxisome proliferator-activated receptor type α; *sirt1*, sirtuin 1; *slc16a1*, monocarboxylate transporter 1; *slc16a7*, monocarboxylate transporter 2; *slc27a1*, fatty acid transport protein 1; *slc2a2*, glucose facilitated transporter type 2.*

### Western Blot Analysis

Frozen samples (20 mg) were homogenized in 1 ml of buffer containing 150 mM NaCl, 10 mM Tris–HCl, 1 mM EGTA, 1 mM EDTA (pH 7.4), 100 mM sodium fluoride, 4 mM sodium pyrophosphate, 2 mM sodium orthovanadate, 1% Triton X-100, 0.5% NP40-IGEPAL, and 1.02 mg ml^–1^ protease inhibitor cocktail (Sigma). Tubes were kept on ice during the whole process to prevent protein denaturation. Homogenates were centrifuged at 1000 × *g* for 15 min at 4°C, and supernatants were again centrifuged at 20,000 × *g* for 30 min. The resulting supernatants were recovered and stored at –80°C. The concentration of protein in each sample was determined using Bradford assay with bovine serum albumin as standard. Protein lysates (10 μg) were Western blotting using appropriate antibodies from 1) Cell Signaling Technology (Leiden, Netherlands): anti-phospho Akt (Ser473) ref. #4060, anti-carboxyl terminal Akt ref. #9272, anti-phospho Ampkα (Thr172) ref. #2531, anti-Ampkα ref. #2532, anti-phospho mTor (Ser2448) ref. #5536, and anti-β-Tubulin ref. #2146, 2) Sigma: anti m-Tor ref. #T2949 or, 3) LifeSpan BioSciences (Seattle, WA, United States): anti-Chrebp ref. #C187144. All these antibodies cross-react successfully with rainbow trout proteins of interest (Skiba-Cassy et al., 2009; Sánchez-Gurmaches et al., 2010; Kamalam et al., 2012; Velasco et al., 2016; Conde-Sieira et al., 2018). After washing, membranes were incubated with an IgG-HRP secondary antibody (Abcam, ref. #2015718) and bands were quantified by Image Lab software version 5.2.1 (BIO-RAD) in a Chemidoc Touch imaging system (BIO-RAD). Samples of Western blots for each protein are shown in Supplementary Material.

### Statistics

Comparisons among groups were carried out with Student *t*-test using the statistical package SigmaStat. Differences were considered statistically significant at *p* < 0.05.

## Results

### Experiment 1

**FIGURE 1:**
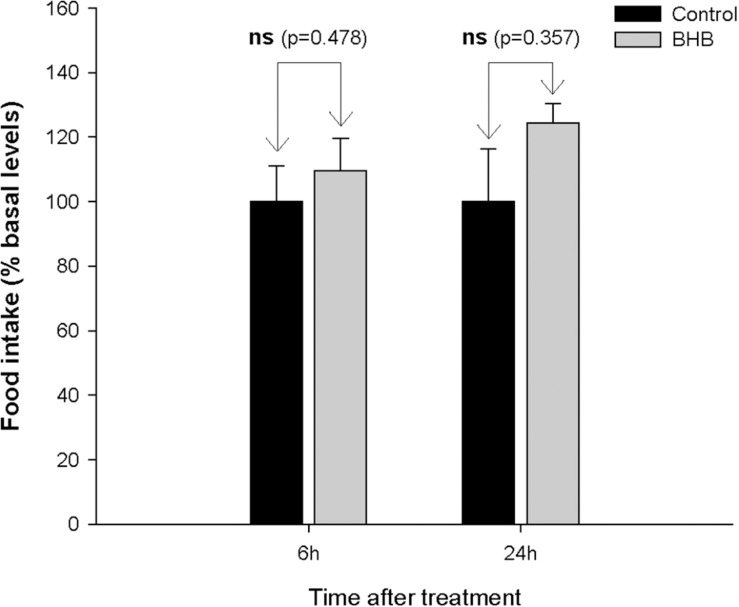
Food intake in rainbow trout 6 and 24 h after intracerebroventricular administration of 1 μL 100 g^– 1^ body mass of saline solution alone (Control) or containing 0.5 μmol of β-hydroxybutyrate (BHB). Food intake is displayed as the percentage of food ingested with respect to baseline levels (calculated as the average of food intake the 3 days previous to experiment) and was normalized to control group (100%). Each value of food intake is the mean ± SEM of 3 experiments (*N* = 3) with *n* = 10 fish per treatment in each tank per experiment. ns, not significant difference.

No significant changes were observed after BHB treatment in food intake (Figure 1).

### Experiment 2

No significant changes were observed after BHB treatment in levels of plasma metabolites (Table 2).

**TABLE 2:** Levels of metabolites (mM) in plasma of rainbow trout 6 h after intracerebroventricular administration of 1 μL 100 g^–1^ body mass of saline solution alone (control) or containing 0.5 μmol of β-hydroxybutyrate (BHB).

	**Treatment**		
**Parameter**	**Control**	**BHB**	***p*-value**
Acetoacetate	0.13 ± 0.09	0.13 ± 0.03	0.185
Fatty acid	0.28 ± 0.03	0.32 ± 0.02	0.218
Glucose	6.53 ± 0.44	6.67 ± 0.35	0.812
β-hydroxybutyrate	0.24 ± 0.01	0.22 ± 0.01	0.222
Lactate	2.47 ± 0.40	2.26 ± 0.34	0.756

*Each value is the mean ± SEM of *n* = 22 fish per treatment.*

The effects of BHB treatment on the levels of metabolites, enzyme activities and mRNA abundance of transcripts in hypothalamus is shown in Table 3. BHB treatment decreased levels of lactate and mRNA abundance of *slc16a7, slc27a1, hadh*, and *pck1* without affecting the remaining parameters assessed.

**TABLE 3:** Levels of metabolites, enzyme activities, and relative mRNA abundance of transcripts in hypothalamus of rainbow trout 6 h after intracerebroventricular administration of 1 μL 100 g^–1^ body mass of saline solution alone (control) or containing 0.5 μmol of β-hydroxybutyrate (BHB).

			**Treatment**		
**Category**	**Type**	**Parameter**	**Control**	**BHB**	***p*-value**
Metabolite levels		Glucose	0.97 ± 0.46	0.91 ± 0.28	0.904
(μmol g^–1^)		Lactate	5.90 ± 0.31	4.93 ± 0.31**^∗^**	0.044
		β-hydroxybutyrate	0.90 ± 0.17	0.96 ± 0.08	0.860
		Acetoacetate	2.79 ± 0.44	3.26 ± 0.50	0.498
		BHB/acetoacetate	0.32 ± 0.15	0.29 ± 0.09	0.786
		Fatty acid	0.15 ± 0.01	0.13 ± 0.03	0.205
Enzyme activities	Ketone body metabolism	β-HBDH	1.24 ± 0.67	1.95 ± 1.49	0.959
(mU ⋅ mg^–1^ protein)		ACAT	100.9 ± 5.9	105.6 ± 7.8	0.637
		SCOT	22.8 ± 1.20	20.8 ± 3.10	0.620
mRNA abundance	Neuropeptides	*agrp1*	1.00 ± 0.26	0.43 ± 0.07	0.352
		*cartpt*	1.00 ± 0.18	0.71 ± 0.10	0.136
		*npy*	1.00 ± 0.11	1.04 ± 0.19	0.839
		*pomca1*	1.00 ± 0.23	0.76 ± 0.12	0.414
	Transcription factors	*bsx*	1.00 ± 0.15	0.76 ± 0.11	0.223
		*creb1*	1.00 ± 0.19	0.85 ± 0.13	0.527
		*foxo1*	1.00 ± 0.14	0.63 ± 0.10	0.070
		*ppara*	1.00 ± 0.11	1.22 ± 0.12	0.252
	Integrative sensors	*mtor*	1.00 ± 0.14	0.70 ± 0.09	0.135
		*sirt1*	1.00 ± 0.03	0.90 ± 0.03	0.064
	Ketone body metabolism	*bdh1*	1.00 ± 0.23	0.69 ± 0.08	0.205
		*hmgcs2*	1.00 ± 0.05	0.97 ± 0.19	0.898
	Monocarboxylate transport	*slc16a1*	1.00 ± 0.08	0.89 ± 0.19	0.567
		*slc16a7*	1.00 ± 0.08	0.72 ± 0.09**^∗^**	0.049
	Lactate metabolism	*ldha*	1.00 ± 0.26	0.66 ± 0.15	0.276
		*ldhb*	1.00 ± 0.10	0.82 ± 0.12	0.305
	K_ATP_ channel	*abcc8*	1.00 ± 0.15	0.68 ± 0.07	0.081
		*kcnj11*	1.00 ± 0.10	0.97 ± 0.16	0.863
	Fatty acid transport	*cd36*	1.00 ± 0.09	0.84 ± 0.15	0.370
		*slc27a1*	1.00 ± 0.15	0.57 ± 0.07**^∗^**	0.033
	Lipolysis	*cpt1c*	1.00 ± 0.14	0.92 ± 0.13	0.694
		*hadh*	1.00 ± 0.10	0.68 ± 0.08**^∗^**	0.031
	Glycolysis	*gck*	1.00 ± 0.16	0.86 ± 0.13	0.512
	Gluconeogenesis	*pck1*	1.00 ± 0.12	0.46 ± 0.08**^∗^**	0.010

*Each value is the mean ± SEM of *n* = 10 (metabolite and enzyme activities) or *n* = 6 (mRNA abundance) fish per treatment. Data of mRNA abundance are referred to the control group (results were previously normalized by *actb* and *eef1a1* mRNA levels, which did not show changes among groups). ^∗^Significantly different (*p* < 0.05) from control group.*

**FIGURE 2:**
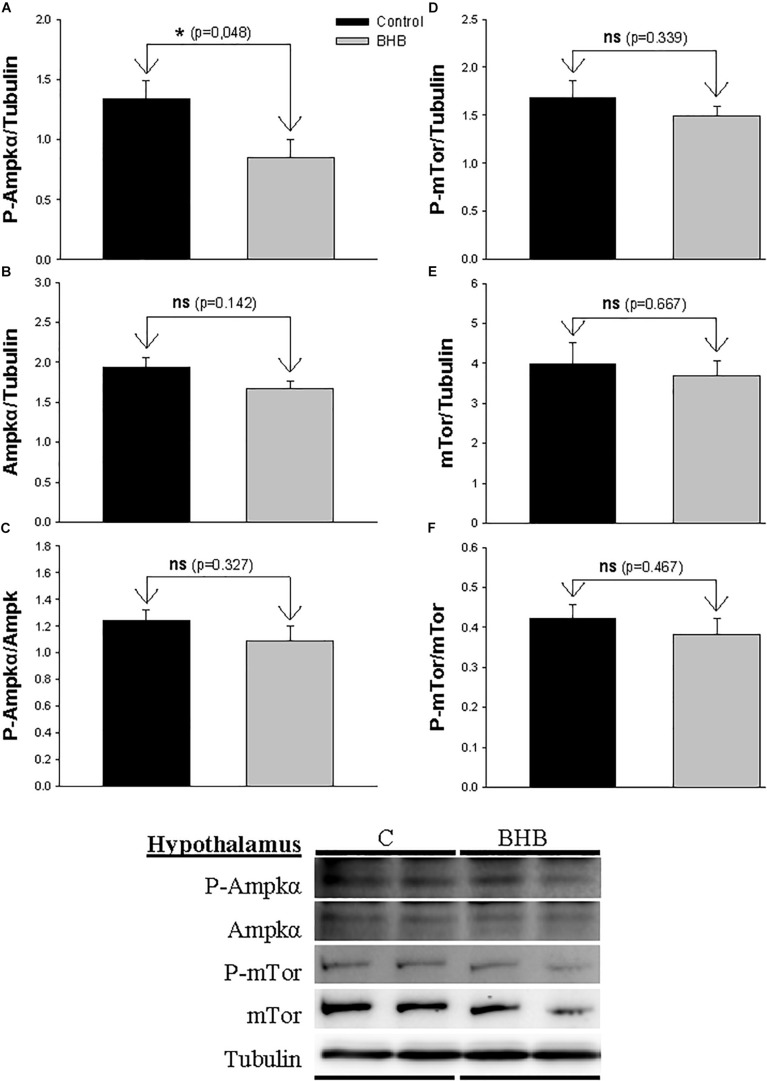
Western blot analysis of Ampkα **(A–C)** and mTor **(D–F)** protein levels in hypothalamus of rainbow trout 6 h after intracerebroventricular administration of 1 μL 100 g^– 1^ body mass of saline solution alone (Control) or containing 0.5 μmol of β-hydroxybutyrate (BHB). Ten micrograms of total protein were loaded on the gel per lane. Western blots were performed on six individual samples per treatment, and representative blots are shown. Graphs represent the amount of phosphorylated protein **(A,D)**, the total amount of the target protein **(B,E)** and the ratio between the phosphorylated protein and the total amount of the target protein **(C,F)**. Each value is the mean + SEM of *n* = 6 fish per treatment. ns, not significant difference. ^∗^ indicates significant differences (*p* < 0.05) compared to control group.

The levels and phosphorylation status of Ampkα and Mtor in hypothalamus is shown in Figure 2. The levels of the phosphorylated form of Ampkα decreased after BHB treatment without affecting the non-phosphorylated form. mTor was not affected by treatment.

The effects of BHB treatment on the levels of metabolites, enzyme activities and mRNA abundance of transcripts in hindbrain is shown in Table 4. BHB treatment decreased levels of lactate, and mRNA abundance of *ldha* and *slc27a1* whereas an increase occurred in acetoacetate levels without affecting the remaining parameters assessed.

**TABLE 4:** Levels of metabolites, enzyme activities, and relative mRNA abundance of transcripts in hindbrain of rainbow trout 6 h after intracerebroventricular administration of 1 μL 100 g^–1^ body mass of saline solution alone (control) or containing 0.5 μmol of β-hydroxybutyrate (BHB).

			**Treatment**		
**Category**	**Type**	**Parameter**	**Control**	**BHB**	***p*-value**
Metabolite levels		Glucose	0.80 ± 0.43	0.62 ± 0.22	0.805
(μmol g^–1^)		Lactate	7.94 ± 0.27	6.59 ± 0.35 **^∗^**	0.009
		β-hydroxybutyrate	0.55 ± 0.13	0.44 ± 0.10	0.513
		Acetoacetate	3.36 ± 0.41	4.50 ± 0.51**^∗^**	0.048
		BHB/acetoacetate	0.16 ± 0.04	0.09 ± 0.03	0.204
		Fatty acid	0.03 ± 0.01	0.02 ± 0.01	0.205
Enzyme activities	Ketone body metabolism	β-HBDH	18.8 ± 6.30	4.30 ± 1.90	0.146
(mU ⋅ mg^–1^ protein)		ACAT	106.1 ± 3.90	84.7 ± 10.6	0.112
		SCOT	14.3 ± 4.00	19.1 ± 3.2	0.301
mRNA abundance	Neuropeptides	*agrp1*	1.00 ± 0.27	0.80 ± 0.12	0.818
		*cartpt*	1.00 ± 0.10	0.88 ± 0.13	0.491
		*npy*	1.00 ± 0.16	0.83 ± 0.18	0.569
		*pomca1*	1.00 ± 0.19	0.73 ± 0.15	0.329
	Transcription factors	*bsx*	1.00 ± 0.47	1.70 ± 1.40	0.931
		*creb1*	1.00 ± 0.09	1.45 ± 0.23	0.098
		*foxo1*	1.00 ± 0.12	0.76 ± 0.03	0.121
		*ppara*	1.00 ± 0.22	0.81 ± 0.14	0.496
	Integrative sensors	*mtor*	1.00 ± 0.05	0.87 ± 0.10	0.284
		*sirt1*	1.00 ± 0.08	0.67 ± 0.08	0.072
	Ketone body metabolism	*bdh1*	1.00 ± 0.22	0.65 ± 0.03	0.160
		*hmgcs2*	1.00 ± 0.14	1.03 ± 0.11	0.897
	Monocarboxylate transport	*slc16a1*	1.00 ± 0.17	1.04 ± 0.09	0.827
		*slc16a7*	1.00 ± 0.07	1.21 ± 0.11	0.133
	Lactate metabolism	*ldha*	1.00 ± 0.16	0.30 ± 0.07**^∗^**	< 0.001
		*ldhb*	1.00 ± 0.03	0.84 ± 0.13	0.317
	K_ATP_ channel	*abcc8*	1.00 ± 0.06	1.03 ± 0.21	0.877
		*kcnj11*	1.00 ± 0.18	0.84 ± 0.12	0.452
	Fatty acid transport	*cd36*	1.00 ± 0.10	0.90 ± 0.08	0.468
		*slc27a1*	1.00 ± 0.13	0.45 ± 0.08**^∗^**	0.005
	Lipolysis	*cpt1c*	1.00 ± 0.06	1.02 ± 0.10	0.837
		*hadh*	1.00 ± 0.08	0.81 ± 0.09	0.134
	Gluconeogenesis	*pck1*	1.00 ± 0.06	0.93 ± 0.10	0.582

*Each value is the mean ± SEM of *n* = 10 (metabolite and enzyme activities) or *n* = 6 (mRNA abundance) fish per treatment. Data of mRNA abundance are referred to the control group (results were previously normalized by *actb* and *eef1a1* mRNA levels, which did not show changes among groups). ^∗^Significantly different (*p* < 0.05) from control group.*

**FIGURE 3:**
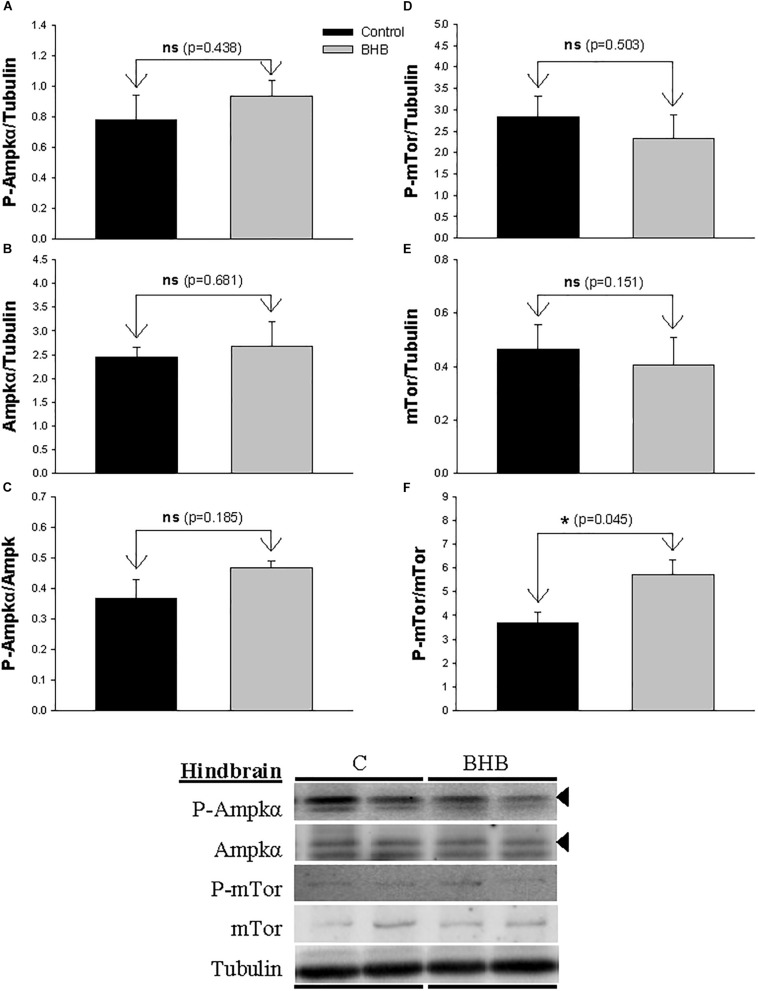
Western blot analysis of Ampkα **(A–C)** and mTor **(D–F)** protein levels in hindbrain of rainbow trout 6 h after intracerebroventricular administration of 1 μL 100 g^– 1^ body mass of saline solution alone (Control) or containing 0.5 μmol of β-hydroxybutyrate (BHB). Ten micrograms of total protein were loaded on the gel per lane. Western blots were performed on six individual samples per treatment, and representative blots are shown. Graphs represent the amount of phosphorylated protein **(A,D)**, the total amount of the target protein **(B,E)** and the ratio between the phosphorylated protein and the total amount of the target protein **(C,F)**. Each value is the mean + SEM of *n* = 6 fish per treatment. ns, not significant difference. ^∗^ indicates significant differences (*p* < 0.05) compared to control group.

The levels and phosphorylation status of Ampkα and mTor in hindbrain is shown in Figure 3. The ratio of phosphorylated vs. non-phosphorylated mTor increased after BHB treatment without affecting levels of both forms separately. The levels and phosphorylation status of Ampkα were not affected by treatment.

The effects of BHB treatment on the levels of metabolites, enzyme activities and mRNA abundance of transcripts in liver is shown in Table 5. BHB treatment decreased the activities of GCK and PK, and mRNA abundance of *gys1* whereas an increase occurred in mRNA abundance of *g6pc, fbp1, cpt1a, glud1*, and *gpt*. No significant changes occurred in the remaining parameters assessed.

**TABLE 5:** Levels of metabolites, enzyme activities, and relative mRNA abundance of transcripts in liver of rainbow trout 6 h after intracerebroventricular administration of 1 μL 100 g^–1^ body mass of saline solution alone (control) or containing 0.5 μmol of β-hydroxybutyrate (BHB).

			**Treatment**		
**Category**	**Type**	**Parameter**	**Control**	**BHB**	***p*-value**
Metabolite levels		Glucose	17.3 ± 1.6	19.0 ± 1.31	0.424
(μmol g^–1^)		Lactate	1.42 ± 0.16	1.79 ± 0.23	0.210
		Glycogen (glycosyl units)	94.4 ± 25.8	104.1 ± 30.6	0.810
		Fatty acid	0.04 ± 0.01	0.05 ± 0.01	0.831
		Triglyceride	1.28 ± 0.08	1.15 ± 0.10	0.325
		α-amino acid	460.1 ± 14.1	499.9 ± 19.6	0.120
Enzyme activities	Glycolysis	GCK	21.2 ± 1.70	17.4 ± 0.81**^∗^**	0.045
(mU ⋅ mg^–1^ protein)		PK	42.4 ± 2.50	35.1 ± 2.91**^∗^**	0.044
	Gluconeogenesis	G6Pase	13.1 ± 0.30	11.8 ± 0.80	0.279
		PEPCK	9.91 ± 0.38	8.92 ± 0.37	0.082
	Glycogenesis	% GSase a	17.0 ± 4.1	10.2 ± 3.20	0.140
	Lipogenesis	FAS	1.39 ± 0.22	1.82 ± 0.44	0.672
	Lipolysis	CPT-1	1.34 ± 0.29	1.45 ± 0.16	0.758
	Amino acid catabolism	GDH	6.58 ± 0.58	6.64 ± 0.33	0.930
		ALT	6.10 ± 0.23	5.60 ± 0.32	0.228
	Ketone body metabolism	β-HBDH	0.15 ± 0.01	0.13 ± 0.01	0.383
		ACAT	106.3 ± 11.9	113.7 ± 9.9	0.643
mRNA abundance	Glycolysis	*gck*	1.00 ± 0.52	0.26 ± 0.09	0.212
	Gluconeogenesis	*fbp1*	1.00 ± 0.36	2.41 ± 0.44**^∗^**	0.040
		*g6pc*	1.00 ± 0.05	1.39 ± 0.16**^∗^**	0.043
		*pck1*	1.00 ± 0.16	1.13 ± 0.10	0.433
	Glycogenesis	*gys1*	1.00 ± 0.17	0.54 ± 0.14**^∗^**	0.041
	Glucose transport	*slc2a2*	1.00 ± 0.05	1.14 ± 0.07	0.111
	Lipogenesis	*fasn*	1.00 ± 0.17	0.83 ± 0.20	0.528
	Lipolysis	*cpt1a*	1.00 ± 0.31	2.49 ± 0.49**^∗^**	0.037
	Amino acid catabolism	*glud1*	1.00 ± 0.17	1.64 ± 0.24**^∗^**	0.048
		*gpt*	1.00 ± 0.17	1.53 ± 0.14**^∗^**	0.041
	Ketone body metabolism	*bdh1*	1.00 ± 0.40	0.56 ± 0.17	0.352
		*hmgcs2*	1.00 ± 0.34	0.96 ± 0.30	0.932

*Each value is the mean ± SEM of *n* = 10 (metabolite and enzyme activities) or *n* = 6 (mRNA abundance) fish per treatment. Data of mRNA abundance are referred to the control group (results were previously normalized by *actb* and *eef1a1* mRNA levels, which did not show changes among groups). ^∗^Significantly different (*p* < 0.05) from control group.*

**FIGURE 4:**
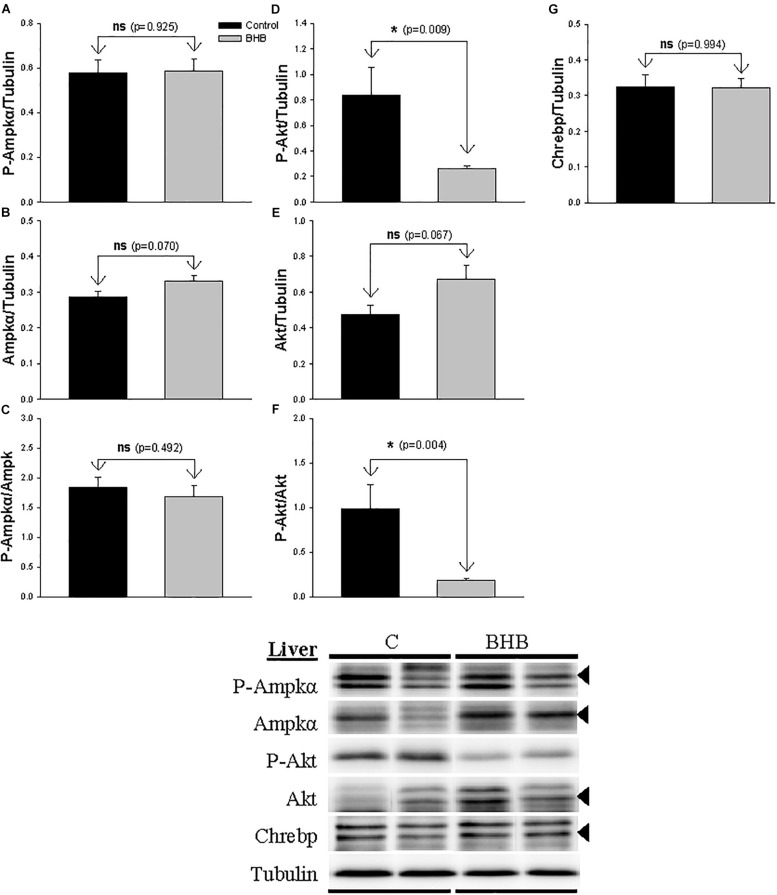
Western blot analysis of Ampkα **(A–C)**, Akt **(D–F)** and Chrebp **(G)** protein levels in liver of rainbow trout 6 h after intracerebroventricular administration of 1 μL 100 g^– 1^ body mass of saline solution alone (Control) or containing 0.5 μmol of β-hydroxybutyrate (BHB). Ten micrograms of total protein were loaded on the gel per lane. Western blots were performed on six individual samples per treatment, and representative blots are shown. Graphs represent the amount of phosphorylated protein **(A,D)**, the total amount of the target protein **(B,E,G)** and the ratio between the phosphorylated protein and the total amount of the target protein **(C,F)**. Each value is the mean + SEM of *n* = 6 fish per treatment. ns, not significant difference. ^∗^ indicates significant differences (*p* < 0.05) compared to control group.

The levels and phosphorylation status of proteins of interest in liver is shown in Figure 4. The levels of P-Akt and P-Akt/akt decreased after BHB treatment while no significant changes occurred in the levels of Akt. Finally, no significant changes occurred in the levels and phosphorylation status of Ampk and in the levels of Chrebp.

## Discussion

### The Presence of BHB in the Brain Does Not Change Food Intake

No prior studies in fish assessed the effect of KB on food intake. Available studies in fish only evaluated levels of KB in plasma and brain in food deprived fish (Soengas et al., 1996; Figueroa et al., 2000; Polakof et al., 2007). This lack of effect in food intake is different than that observed in mammals where increased (Iwata et al., 2011; Carneiro et al., 2016a, b) or decreased (Le Foll et al., 2014, 2015) food intake occurred after BHB treatment. However, we should compare our results with those in mammals with caution since different approaches were used in mammals to assess the relationship of KB with food intake (Le Foll et al., 2014, 2015; Carneiro et al., 2016a, b). In any case, the present results allow us to hypothesize that BHB might not relate to food intake control in fish when administered centrally, a response different to that known in mammals. Since the time period in which changes in KB occurred during food deprivation in fish is considerably longer than in mammals, we cannot exclude the possibility that changes in food intake might occur after prolonged BHB treatment.

### The Presence of BHB in the Brain Elicits Few Changes in Parameters Putatively Related to Ketone Sensing and Not Affected Food Intake Regulation

β-hydroxybutyrate treatment did not alter BHB levels in brain regions assessed suggesting a quick metabolization of this KB. Both brain areas display a different behavior regarding levels of KB as demonstrated by the raise in acetoacetate levels in hindbrain but not in hypothalamus after BHB treatment, and BHB/acetoacetate ratio, which was lower in hindbrain than in hypothalamus under control conditions. We have no explanation for this interesting difference in behavior between both regions.

The ICV treatment with BHB elicited changes in brain energy metabolism as demonstrated by decreased potential of gluconeogenesis (decreased mRNA abundance of *pck1*) in hypothalamus as well as decreased capacity for transport of fatty acid both in hypothalamus and hindbrain (decreased mRNA abundance of *slc27a1*) and decreased use of fatty acid in hypothalamus (decreased mRNA abundance of *hadh*). The effect on fatty acid is probably due to the lack of necessity for taking them up to act as precursors of KB. The metabolism of lactate is also apparently inhibited in both brain areas, as demonstrated reduced lactate levels and reduced mRNA abundance of *ldha* and *slc16a7*. These changes reflect a decreased necessity of using glucose, lactate, and fatty acid for fuel purposes in the brain, which is logical considering the presence of an alternative fuel such as KB, and are comparable to those described in mammals (Rojas-Morales et al., 2016; Newman and Verdin, 2017). There are some differences when comparing the response of hypothalamus and hindbrain but in general they are comparable.

Besides direct metabolic effects, the presence of KB within the brain detected by ketone-sensing mechanisms is expected to act as a signal of nutrient abundance within the brain inducing changes in the modulation of food intake (Le Foll et al., 2014). A first candidate to function as a ketone sensor would be the metabolism of KB. We have not observed any change in parameters related to these pathways such as activities of β-BHDH, SCOT and ACAT, as well as mRNA abundance of *bdh1* and *hmgcs2*. In mice, Carneiro et al. (2016b) did not observe changes in β-HBDH or HMGCS activities in hypothalamus after BHB central infusion. However, in rat Le Foll et al. (2015) observed a reverted effect of food intake after HMGCS inhibition in brain. These key enzymes are regulated by different proteins and transcription factors such as sirtuin1, mTOR or PPARα (Newman and Verdin, 2017). The absence of changes in mRNA abundance of these transcription factors in the present study is therefore in agreement with the lack of changes in KB metabolism.

Transport of KB through monocaboxylate carriers (MCTs) could be another putative ketone sensor (Laeger et al., 2012). Of the two carriers involved in the monocarboxylate transport into the brain (MCT1 and MCT2), also present in rainbow trout brain (Omlin and Weber, 2013), we observed a decrease in the mRNA abundance of *slc16a7* (corresponding to MCT2) after BHB treatment in hypothalamus. This effect is comparable to that observed in hypothalamus of either food deprived (Schutkowski et al., 2014) or BHB-infused (Carneiro et al., 2016b) mice. MCT2 is supposed to be present in neurons for the uptake of monocarboxylates while MCT1 is involved in their release (Laeger et al., 2010). However, Carneiro et al. (2016a) observed no changes after BHB infusion in mRNA abundance of *Mct1, Mct2*, and *Mct4* in mice.

We observed a decrease in levels of P-Ampkα in hypothalamus of BHB-treated fish. Such a decrease is compatible with the role of AMPKα as an energy sensor being inactivated under increased levels of energy within the cell (López et al., 2016; López, 2017). In mice, the infusion of BHB resulted in an increase in the ratio P-AMPK/AMPK (Carneiro et al., 2016a). Considering that in mice an increase in food intake occurred in parallel with changes in mRNA abundance of *Npy*, *Agrp, Pomc*, and *Cart*, this is suggesting clearly different responses between both vertebrate groups. BHB treatment *in vitro* in hypothalamic cell lines (Laeger et al., 2012) resulted in increased or decreased P-AMPK/AMPK ratio with low or high glucose concentrations, respectively. The specificity of the effect of hypothalamic Ampkα in the present study is reinforced by the lack of changes noticed in the other brain area assessed (hindbrain) as well as by the lack of changes in the levels and phosphorylation status of mTor. However, it is not clear why the decrease in P-Ampkα did not induce in brain a downstream change in the pathways involved in the regulation of food intake. This can relate to the absence of significant changes in P-Ampkα/Ampkα ratio and/or to the existence of additional pathways involved.

In hypothalamus, when nutrient levels rise, a decrease in the levels of BSX and CREB as well as an increase in those of FoxO1 result in the inhibition of mRNA abundance of *Agrp* and *Npy*, and the enhancement of *Cartpt* and *Pomc*, resulting in a decrease in food intake as demonstrated in mammals (Nogueiras et al., 2008; Diéguez et al., 2011; Varela et al., 2011) with some evidence also existing in fish (Conde-Sieira et al., 2018). However, in the present study, we have not observed any significant change in mRNA abundance of *bsx, creb1*, and *foxo1* in hypothalamus and hindbrain of fish treated with BHB. The absence of changes in these transcription factors is also in agreement with the lack of changes in the mRNA abundance of the neuropeptides under their control involved in the metabolic regulation of food intake such as *agrp1, npy, pomca1*, and *cartpt*. This is in contrast with the mammalian model where mRNA abundance of the orexigenic neuropeptides *Agrp* and *Npy* increase after BHB treatment without affecting *Pomc* and *Cartpt* (Iwata et al., 2011; Laeger et al., 2012; Carneiro et al., 2016a, b), and this was concomitant with changes in food intake (Carneiro et al., 2016a) not observed in rainbow trout. The absence of changes in neuropeptide mRNA abundance is consistent with the lack of changes observed in food intake of BHB-treated fish.

### Liver Metabolism Was Affected by Central KB Treatment

The presence of KB in the brain is able to induce changes in liver metabolism of mammals, at least in diabetic rats (Park et al., 2011). In rainbow trout, central BHB treatment affected several metabolic pathways in the liver. Since no changes occurred in plasma levels of metabolites, it seems reasonable that the hepatic effects are the result of a signal arriving to the liver from the brain, and not the result of changes in plasma metabolites. In this way, downstream pathways through which hypothalamus/hindbrain modulate hepatic metabolism might be based on sympathetic and parasympathetic systems that innervate gastrointestinal tract and liver in the same species (Seth and Axelsson, 2010). Accordingly, a functional relationship between central presence of nutrients and peripheral effects on liver metabolism comes from previous studies in the same species in which metabolic changes in liver occurred after ICV treatment with glucose, lactate, oleate or octanoate (Polakof and Soengas, 2008; Librán-Pérez et al., 2014) or after central inhibition of AMPKα2 (Conde-Sieira et al., 2019). This signal might relate to the inhibition of Ampkα in hypothalamus and/or activation of mTor in hindbrain. However, we cannot exclude the possibility that metabolic changes in liver be the result of a hormone being released by the brain.

Ketone body metabolism in liver was not apparently affected by central BHB treatment in agreement with studies carried out in mice at comparable times (Carneiro et al., 2016a). In contrast, central administration of BHB clearly affected glucose metabolism in the liver of rainbow trout. A clear decrease in the capacity of this tissue to use glucose as a fuel was supported by the decrease observed in GCK activity and *gck* mRNA abundance (though the later was not significant) as well as in PK activity. Moreover, a clear decrease in the capacity of this tissue to synthesize glycogen was also evident based on decreased mRNA abundance of *gys1* and the non-significant decrease in % GSase a activity. mRNA abundance of parameters involved in gluconeogenesis displayed an intriguing increase as demonstrated values of *fbp1* and *g6pc* and the non-significant increase of *pck1*. A hypothetical increase in gluconeogenic capacity would be contrary to that known in mammals where gluconeogenesis was reduced in liver after central BHB infusion (Carneiro et al., 2016a, b). Is this tempting to suggest that the presence of KB in the brain elicited a signal in liver indicative that no necessity of using additional fuels is necessary thus favoring anabolic instead of catabolic pathways. This would match with a raised potential of gluconeogenesis. A rise in gluconeogenic capacity in liver of this species is also evident under conditions of food deprivation in which increased levels of KB are present and used in brain simultaneously (Soengas et al., 1998). We may suggest that gluconeogenic potential activated in rainbow trout liver by a signal coming from the brain informing of the presence of KB in a way comparable to that occurring under food deprivation conditions. It is relevant to mention however that no changes occurred in the levels of glucose in plasma and liver. This absence of changes is comparable to that observed in rat after ICV BHB treatment (Arase et al., 1988) as well as in rainbow trout after 24 h of food deprivation (Figueroa et al., 2000). The impact of ICV BHB treatment in liver glucose metabolism in rat appears to be dependent on insulin signaling (Park et al., 2011) through Akt phosphorylation and reduced expression of *pck1*. In the present study, we observed reduced Akt phosphorylation and increased gluconeogenic potential after BHB treatment, i.e., a response apparently opposed to that of mammals. Akt reduces its activity in liver in response to KB (Rojas-Morales et al., 2016). We observed the same effect, perhaps related to inhibition of insulin signaling. In fact, results obtained in liver are highly coherent with decreased insulin signaling. Thus, reduced pAkt could be the consequence of reduced insulin levels, which would reduce glucose oxidation capacity (coincident with decreased GCK activity), and release the inhibitory effect on gluconeogenesis (actually increased in the present study). The reduced potential of β-oxidation is also compatible with low insulin levels.). In rat Park et al. (2011) observed that a 4-week central infusion of BHB potentiated insulin signaling in liver. This difference could be the result of differences between long- and short-term treatment of BHB not evaluated in the present study.

In BHB-treated fish the lipogenic potential was unaffected (no changes in *fasn* mRNA abundance and FAS activity) while that of fatty acid oxidation increased (increased *cpt1a* mRNA abundance) as well as that of amino acid catabolism (increased *glud1* and *gpt* mRNA abundance). On the other hand, the increased potential of β-oxidation could result in more available fatty acids for ketogenesis. However, some caution must be taken since no significant changes occurred in the activities of CPT-1 and GDH. Thus, liver apparently enhanced the potential for use of amino acid and lipid as energy source instead of glucose, which could relate to the fact that the signal elicited by KB in the brain is informing of glucose scarcity. This is not surprising, from the teleost fish point of view, considering the relative low importance of glucose metabolism (except in brain) in this group compared with that of amino acid and lipid (Polakof et al., 2011, 2012) but highlights a difference again with mammals where no comparable changes occurred.

### Perspectives and Significance

We assessed, for the first time in teleost fish, the impact of central treatment with KB on energy homeostasis through regulation of food intake and peripheral metabolism. The presence of BHB in the brain did not result in changes in food intake. This might relate to the absence of major changes in the cascade of events from the detection of KB through ketone-sensing mechanisms, changes in transcription factors, and changes in the mRNA abundance of neuropeptides regulating food intake. This response is different than that of mammals. The differences between both groups might relate to the reduced importance of KB for brain metabolism of teleost fish compared with mammals (Soengas et al., 1998), which also relates to the known resistance of teleost fish to the effects of food deprivation (Navarro and Gutiérrez, 1995). In contrast, central administration of BHB, as a signal of metabolic shortage, induced changes in liver energy metabolism. These can be summarized in a decreased use of glucose and probably in increased potential for the use of amino acid and lipid instead of glucose. These metabolic conditions are comparable to those occurring under food deprivation conditions in the same species but different to those of mammals under similar treatments.

## Data Availability

The datasets generated for this study are available on request to the corresponding author.

## Ethics Statement

The animal study was reviewed and approved by the experiments described comply with the ARRIVE Guidelines, and were carried out in accordance with the guidelines of the European Union Council (2010/63/UE), and of the Spanish Government (RD 53/2013) for the use of animals in research, and were approved by the Ethics Committee of the Universidade de Vigo.

## Author Contributions

SC, JM, and JS contributed to conception and design of the research. SC, CV, CO-R, and MC-S performed the experiments and analyzed the data. SC and JS prepared the figures. All authors interpreted the results of experiments, edited and revised the manuscript, and drafted the manuscript. JS approved the final version of manuscript.

## Conflict of Interest Statement

The authors declare that the research was conducted in the absence of any commercial or financial relationships that could be construed as a potential conflict of interest.

## Footnotes

1 http://bioinfo.ut.ee/primer3-0.4.0/primer3/

## References

[B1] Arase K. Fisler J. S. Shargill N. S. York D. A. Bray G. A. (1988). Intracerebroventricular infusions of 3-OHB and insulin in a rat model of dietary obesity. *Am. J. Physiol. Regul. Integr. Comp. Physiol.* 255 R974–R981. 3059829 10.1152/ajpregu.1988.255.6.R974

[B2] Carneiro L. Geller S. Fioramonti X. Hebert A. Repond C. Leloup C. (2016a). Evidence for hypothalamic ketone bodies sensing: impact on food intake and peripheral metabolic responses in mice. *Am. J. Physiol. Endocrinol. Metab.* 310 E103–E115. 10.1152/ajpendo.00282.2015 26530151

[B3] Carneiro L. Geller S. Hebert A. Repond C. Fioramonti X. Leloup C. (2016b). Hypothalamic sensing of ketone bodies after prolonged cerebral exposure leads to metabolic control dysregulation. *Sci. Rep.* 6:34909. 10.1038/srep34909 27708432 PMC5052612

[B4] Conde-Sieira M. Capelli V. Álvarez-Otero R. Comesaña S. Liñares-Pose L. Velasco C. (2019). Differential role of hypothalamic AMPKα isoforms in fish: an evolutive perspective. *Mol. Neurobiol.* 56 5051–5066. 10.1007/s12035-018-1434-9 30460617

[B5] Conde-Sieira M. Ceinos R. M. Velasco C. Comesaña S. López-Patiño M. A. Míguez J. M. (2018). Response of rainbow trout’s (*Oncorhynchus mykiss*) hypothalamus to glucose and oleate assessed through transcription factors BSX, ChREBP, CREB, and FoxO1. *J. Comp. Physiol. A* 204 893–904. 10.1007/s00359-018-1288-7 30225518

[B6] Conde-Sieira M. Soengas J. L. (2017). Nutrient sensing systems in fish: impact on food intake regulation and energy homeostasis. *Front. Neurosci.* 10:603. 10.3389/fnins.2016.00603 28111540 PMC5216673

[B7] Delgado M. J. Cerdá-Reverter J. M. Soengas J. L. (2017). Hypothalamic integration of metabolic, endocrine, and circadian signals in fish: involvement in the control of food intake. *Front. Neurosci.* 11:354. 10.3389/fnins.2017.00354 28694769 PMC5483453

[B8] Diéguez C. Vazquez M. J. Romero A. López M. Nogueiras R. (2011). Hypothalamic control of lipid metabolism: focus on leptin, ghrelin and melanocortins. *Neuroendocrinology* 94 1–11. 10.1159/000328122 21576929

[B9] Figueroa R. I. Rodríguez-Sabarís R. Aldegunde M. Soengas J. L. (2000). Effects of food deprivation on 24h-changes in brain and liver carbohydrate and ketone body metabolism of rainbow trout. *J. Fish Biol.* 57 631–646. 10.1006/jfbi.2000.1341

[B10] Furné M. Morales A. E. Trenzado C. E. García-Gallego M. Hidalgo M. C. Domezain A. (2012). The metabolic effects of prolonged starvation and refeeding in sturgeon and rainbow trout. *J. Comp. Physiol. B* 182 63–76. 10.1007/s00360-011-0596-9 21698525

[B11] Iwata K. Kinoshita M. Yamada S. Imamura T. Uenoyama Y. Tsukamura H. (2011). Involvement of brain ketone bodies and the noradrenergic pathway in diabetic hyperphagia in rats. *J. Physiol. Sci.* 61 103–113. 10.1007/s12576-010-0127-6 21234734 PMC10717331

[B12] Kamalam B. S. Medale F. Kaushik S. Polakof S. Skiba-Cassy S. Panserat S. (2012). Regulation of metabolism by dietary carbohydrates in two lines of rainbow trout divergently selected for muscle fat content. *J. Exp. Biol.* 215 2567–2578. 10.1242/jeb.070581 22786633

[B13] Keppler D. Decker K. (1974). “Glycogen: determination with amyloglucosidase,” in *Methods of Enzymatic Analysis*, ed. Bergmeyer H. U. (Cambridge, MA: Academic Press), 1127–1131.

[B14] Laeger T. Metges C. C. Kuhla B. (2010). Role of β-hydroxybutyric acid in the central regulation of energy balance. *Appetite* 54 450–455. 10.1016/j.appet.2010.04.005 20416348

[B15] Laeger T. Pöhland R. Metges C. C. Kuhla B. (2012). The ketone body β-hydroxybutyric acid influences agouti-related peptide expression via AMP-activated protein kinase in hypothalamic GT1-7 cells. *J. Endocrinol.* 213 193–203. 10.1530/JOE-11-0457 22357971

[B16] Langhans W. Wiesenreiter F. Scharrer E. (1983). Different effects of subcutaneous D,L-3-hydroxybutyrate and acetoacetate injections on food intake in rats. *Physiol. Behav.* 31 483–486. 10.1016/0031-9384(83)90070-7 6657769

[B17] Le Foll C. Dunn-Meynel A. A. Miziorko H. M. Levin B. E. (2014). Regulation of hypothalamic neuronal sensing and food intake by ketone bodies and fatty acids. *Diabetes* 63 1259–1269. 10.2337/db13-1090 24379353 PMC3964505

[B18] Le Foll C. Dunn-Meynel A. A. Miziorko H. M. Levin B. E. (2015). Role of VMH ketone bodies in adjusting caloric intake to increased dietary fat content in DIO and DR rats. *Am. J. Physiol. Integr. Comp. Physiol.* 308 R872–R878. 10.1152/ajpregu.00015.2015 25786485 PMC4436979

[B19] Le Foll C. Levin B. E. (2016). Fatty acid-induced astrocyte ketone production and the control of food intake. *Am. J. Physiol. Regul. Integr. Comp. Physiol.* 310 R1186–R1192. 10.1152/ajpregu.00113.2016 27122369 PMC4935491

[B20] Librán-Pérez M. Otero-Rodiño C. López-Patiño M. A. Míguez J. M. Soengas J. L. (2014). Central administration of oleate or octanoate activates hypothalamic fatty acid sensing and inhibits food intake in rainbow trout. *Physiol. Behav.* 129 272–279. 10.1016/j.physbeh.2014.02.061 24631300

[B21] López M. (2017). Hypothalamic AMPK: a golden target against obesity? *Eur. J. Endocrinol.* 176 R235–R246. 10.1530/EJE-16-0927 28232370 PMC5425938

[B22] López M. Nogueiras R. Tena-Sempere M. Diéguez C. (2016). Hypothalamic AMPK: a canonical regulator of whole-body energy balance. *Nature Rev. Endocrinol.* 12 421–432. 10.1038/nrendo.2016.67 27199291

[B23] Mellanby J. Williamson D. H. (1974). “Acetoacetate,” in *Methods of Enzymatic Analysis*, ed. Bergmeyer H. U. (Cambridge, MA: Academic Press), 1841–1843.

[B24] Moore S. (1968). Amino acid analysis: aqueous dimethyl sulfoxide as solvent for the ninhydrin reaction. *J. Biol. Chem.* 243 6281–6283. 5723468

[B25] Navarro I. Gutiérrez J. (1995). “Fasting and starvation,” in *Metabolic Biochemistry, Biochemistry and Molecular Biology of Fishes*, Vol. 4 eds Hochachka P. W. Mommsen T. P. (Amsterdam: Elsevier), 393–434.

[B26] Newman J. C. Verdin E. (2017). β-Hydroxybutyrate: a signaling metabolite. *Annu. Rev. Nutr.* 37 51–76. 10.1146/annurev-nutr-071816-064916 28826372 PMC6640868

[B27] Nogueiras R. López M. Lage R. Perez-Tilve D. Pfluger P. Mendieta-Zerón H. (2008). Bsx, a novel hypothalamic factor linking feeding with locomotor activity, is regulated by energy availability. *Endocrinology* 149 3009–3015. 10.1210/en.2007-1684 18308842 PMC2408820

[B28] Omlin T. Weber J.-M. (2013). Exhausting exercise and tissue-specific expression of monocarboxylate transporters in rainbow trout. *Am. J. Physiol. Regul. Integr. Comp. Physiol.* 304 R1036–R1043. 10.1152/ajpregu.00516.2012 23535457 PMC3680756

[B29] Paoli A. Bosco G. Camporesi E. Mangar D. (2015). Ketosis, ketogenic diet and food intake control: a complex relationship. *Front. Physiol.* 6:27. 10.3389/fpsyg.2015.00027 25698989 PMC4313585

[B30] Park S. Kim D. S. Daily J. W. (2011). Central infusion of ketone bodies modulates body weight and hepatic insulin sensitivity by modifying hypothalamic leptin and insulin signalling pathways in type 2 diabetic rats. *Brain Res.* 1401 95–103. 10.1016/j.brainres.2011.05.040 21652033

[B31] Pfaffl M. W. (2001). A new mathematical model for relative quantification in real-time RT-PCR. *Nucleic Acids Res.* 29:e45. 11328886 10.1093/nar/29.9.e45 PMC55695

[B32] Polakof S. Ceinos R. M. Fernández-Durán B. Míguez J. M. Soengas J. L. (2007). Daily changes in parameters of energy metabolism in brain of rainbow trout: dependence of feeding. *Comp. Biochem. Physiol. A* 146 265–273. 10.1016/j.cbpa.2006.10.026 17126577

[B33] Polakof S. Míguez J. M. Soengas J. L. (2008a). Dietary carbohydrates induce changes in glucosensing capacity and food intake of rainbow trout. *Am. J. Physiol. Regul. Integr. Comp. Physiol.* 295 R478–R489. 10.1152/ajpregu.00176.2008 18525014

[B34] Polakof S. Panserat S. Plagnes-Juan E. Soengas J. L. (2008b). Altered dietary carbohydrates significantly affect gene expression of the major glucosensing components in Brockmann bodies and hypothalamus of rainbow trout. *Am. J. Physiol. Regul. Integr. Comp. Physiol.* 295 R1077–R1088. 10.1152/ajpregu.90476.2008 18685066

[B35] Polakof S. Mommsen T. P. Soengas J. L. (2011). Glucosensing and glucose homeostasis: from fish to mammals. *Comp. Biochem. Physiol. B* 160 123–149. 10.1016/j.cbpb.2011.07.006 21871969

[B36] Polakof S. Panserat S. Soengas J. L. Moon T. W. (2012). Glucose metabolism in fish: a review. *J. Comp. Physiol. B* 182 1015–1045. 10.1007/s00360-012-0658-7 22476584

[B37] Polakof S. Soengas J. L. (2008). Involvement of lactate in glucose metabolism and glucosensing function in selected tissues of rainbow trout. *J. Exp. Biol.* 211 1075–1086. 10.1242/jeb.014050 18344481

[B38] Rojas-Morales P. Tapia E. Pedraza-Chaverri J. (2016). β-Hydroxybutyrate: a signaling metabolite in starvation response? *Cell Signal* 28 917–923. 10.1016/j.cellsig.2016.04.005 27083590

[B39] Sánchez-Gurmaches J. Cruz-Garcia L. Gutiérrez J. Navarro I. (2010). Endocrine control of oleic acid and glucose metabolism in rainbow trout (*Oncorhynchus mykiss*) muscle cells in culture. *Am. J. Physiol. Reg. Integr. Comp. Physiol.* 299 R562–R572. 10.1152/ajpregu.00696.2009 20484701

[B40] Schutkowski A. Wege N. Stangl G. I. König B. (2014). Tissue-specific expression of monocarboxylate transporters during fasting in mice. *PLoS One* 9:e112118. 10.1371/journal.pone.0112118 25390336 PMC4229183

[B41] Seth H. Axelsson M. (2010). Sympathetic, parasympathetic and enteric regulation of the gastrointestinal vasculature in rainbow trout (*Oncorhynchus mykiss*) under normal and postprandial conditions. *J. Exp. Biol.* 213 3118–3126. 10.1242/jeb.043612 20802112

[B42] Skiba-Cassy S. Lansard M. Panserat S. Médale F. (2009). Rainbow trout genetically selected for greater muscle fat content display increased activation of liver TOR signaling and lipogenic gene expression. *Am. J. Physiol. Regul. Integr. Comp. Physiol.* 297 R1421–R1429. 10.1152/ajpregu.00312.2009 19710390

[B43] Soengas J. L. Cerdá-Reverter J. M. Delgado M. J. (2018). Central regulation of food intake in fish: an evolutionary perspective. *J. Mol. Endocrinol.* 60 R171–R199. 29467140 10.1530/JME-17-0320

[B44] Soengas J. L. Strong E. F. Andrés M. D. (1998). Glucose, lactate and β-hydroxybutyrate utilization by rainbow trout brain: changes during food deprivation. *Physiol. Zool.* 71 285–293. 10.1086/515925 9634175

[B45] Soengas J. L. Strong E. F. Fuentes J. Veira J. A. R. Andrés M. D. (1996). Food deprivation and refeeding in Atlantic salmon, *Salmo salar*: effects on brain and liver carbohydrate and ketone bodies metabolism. *Fish Physiol. Biochem.* 15 491–511. 10.1007/BF01874923 24194358

[B46] Varela L. Vázquez M. J. Cordido F. Nogueiras R. Vidal-Puig A. Diéguez C. (2011). Ghrelin and lipid metabolism: key partners in energy balance. *J. Mol. Endocrinol.* 46 R43–R63. 10.1677/JME-10-0068 21169422

[B47] Velasco C. Librán-Pérez M. Otero-Rodiño C. López-Patiño M. A. Míguez J. M. Soengas J. L. (2016). Ceramides are involved in the regulation of food intake in rainbow trout (*Oncorhynchus mykiss*). *Am. J. Physiol. Regul. Integr. Comp. Physiol.* 311 R658–R668. 27465737 10.1152/ajpregu.00201.2016

[B48] Williamson D. H. Bates M. W. Page M. A. Krebs H. A. (1971). Activities of enzymes involved in acetoacetate utilization in adult mammalian tissues. *Biochem. J.* 121 41–47. 10.1042/bj1210041 5165621 PMC1176484

[B49] Williamson D. H. Mellanby J. (1974). “D-(-)-3-Hydroxybutyrate,” in *Methods of Enzymatic Analysis*, ed. Bergmeyer H. U. (Cambridge, MA: Academic Press), 1836–1839.

