# Supplementary material for: Central Treatment of Ketone Body in Rainbow Trout Alters Liver Metabolism Without Apparently Altering the Regulation of Food Intake
Source: Front Physiol. 2019 Sep 18;10:1206. doi: 10.3389/fphys.2019.01206 (PMC6759561; doi:10.3389/fphys.2019.01206)

Supplementary material legend. Representative Western blots for the analysis of P-Ampkα, Ampkα, P-mTor, mTor, and tubulin in hypothalamus, hindbrain and liver, and P-Akt, akt, and Chrebp in liver of rainbow trout 6 h after intracerebroventricular administration of 1 μL.100 g-1 body mass of saline solution alone (Control) or containing 0.5 μmol of β-hydroxybutyrate (BHB). Ten micrograms of total protein were loaded on the gel per lane.

P-Ampkα HY

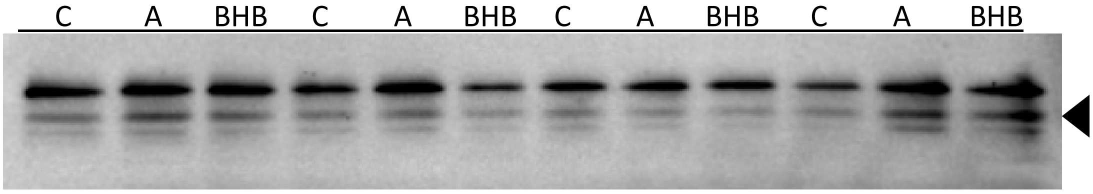

Ampkα HY

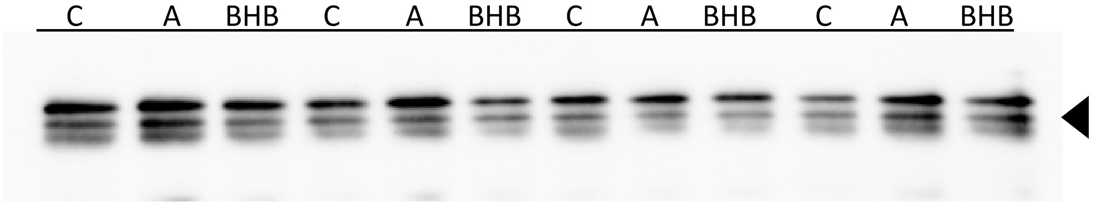

P-Ampkα HB

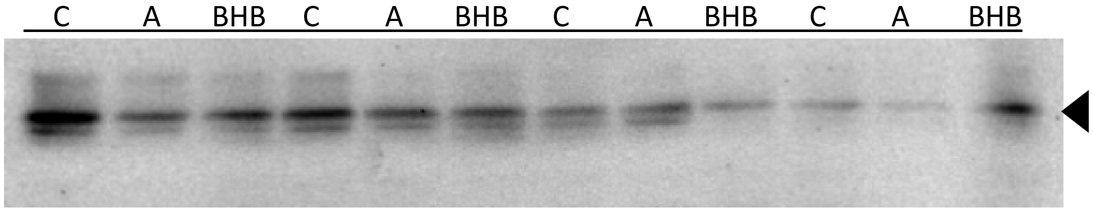

Ampkα HB

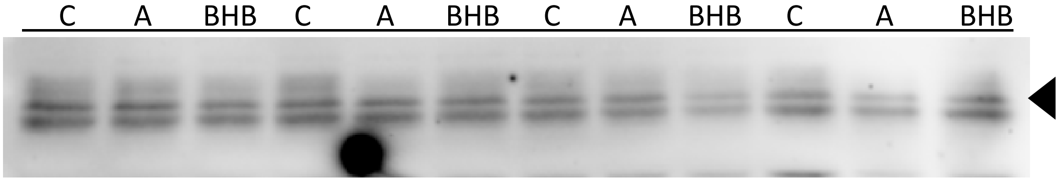

P-Ampkα Liver

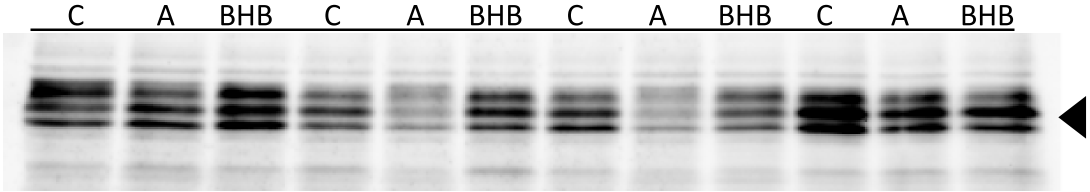

Ampkα Liver

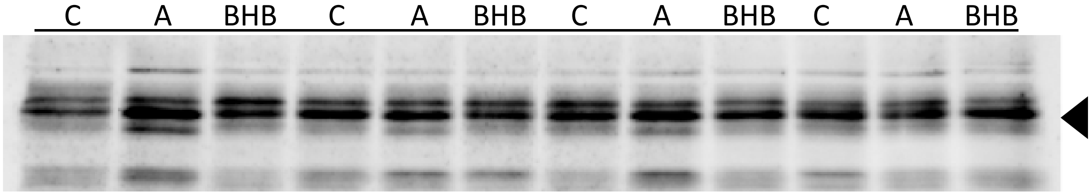

P-Akt Liver

C A BHB C A BHB C A BHB C A BHB

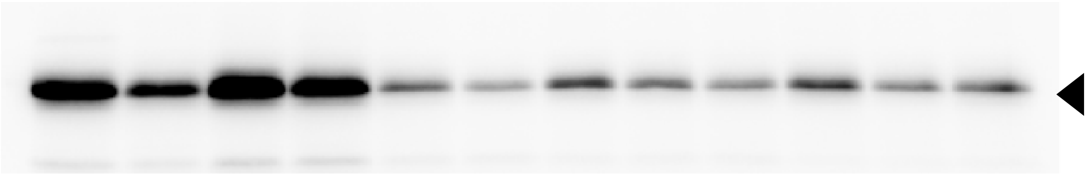

Akt total Liver

C A BHB C A BHB C A BHB C A BHB

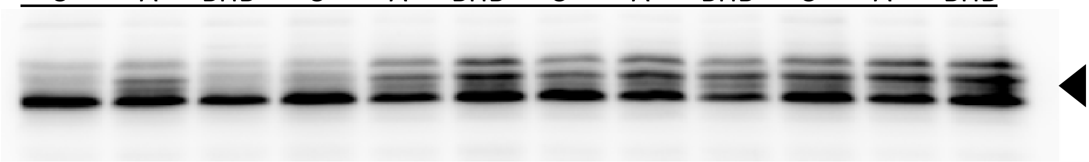

Chrebp Liver

C A BHB C A BHB C A BHB C A BHB

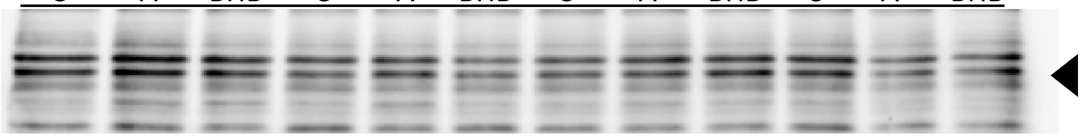

P-mTor HY

C A BHB C A BHB C A BHB C A BHB

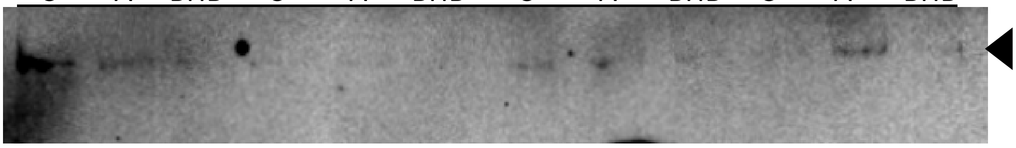

mTor HY

C A BHB C A BHB C A BHB C A BHB

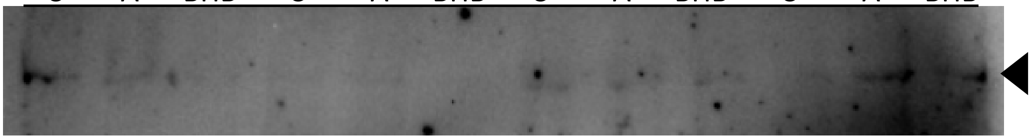

P-mTor HB

C A BHB C A BHB C A BHB C A BHB

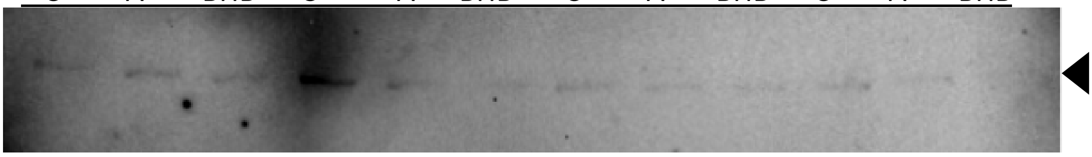

mTor HB

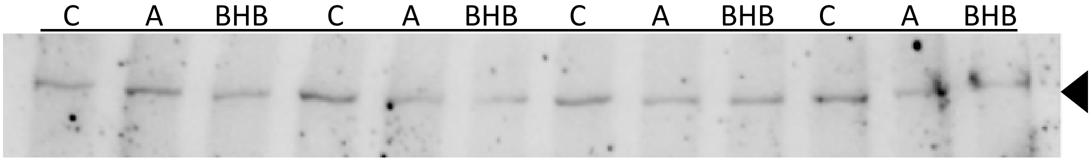

Tubulin HY

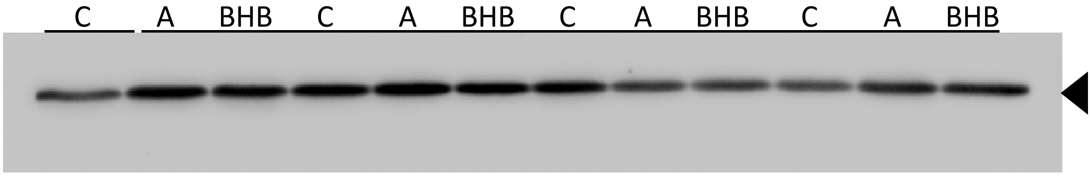

Tubulin HB

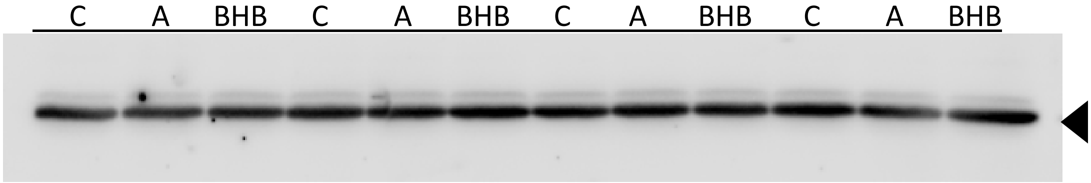

Tubulin Liver

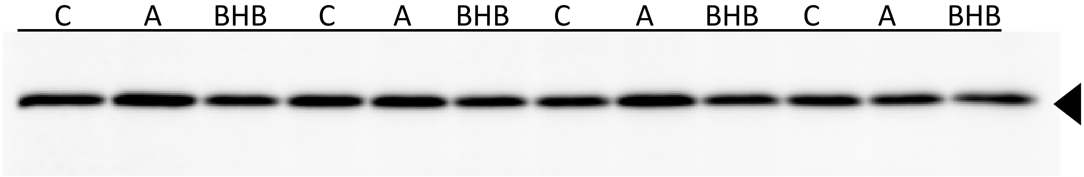

Supplement: Supplementary file 1 [file Image_1.pdf]
